# Homozygous deletions of *UGT2B17* modifies effects of smoking on *TP53-mutations* and relapse of head and neck carcinoma

**DOI:** 10.1186/s12885-015-1220-2

**Published:** 2015-03-31

**Authors:** Aki Mafune, Takanori Hama, Toshihito Suda, Yutaka Suzuki, Masahiro Ikegami, Chikako Sakanashi, Satoko Imai, Akio Nakashima, Takashi Yokoo, Kota Wada, Hiromi Kojima, Mitsuyoshi Urashima

**Affiliations:** 1 Division of Molecular Epidemiology, Jikei University School of Medicine, Tokyo, Japan; 2 Department of Oto-Rhino-laryngology, Jikei University School of Medicine, 3 – 25 - 8 Nishi-shimbashi, Minato-ku, Tokyo, 105-8461 Japan; 3 Department of Pathology, Jikei University School of Medicine, Tokyo, Japan; 4 Department of Surgery, International University of Health and welfare, Tochigi, Japan; 5 Division of Nephrology and Hypertension, Department of Internal Medicine, Jikei University School of Medicine, Tokyo, Japan; 6 Department of Otorhinolaryngology, Toho University, Tokyo, Japan

**Keywords:** UGT2B17, TP53, HNSCC (head and neck squamous-cell carcinoma) and smoking

## Abstract

**Background:**

Smoking induces oncogenic *TP53-mutations* in head and neck squamous cell carcinomas (HNSCCs). Disruptive mutations of *TP53*-gene and expression of *p16* protein [*p16 (+)*] in tumor tissue associate with worse and better prognosis, respectively. UDP-glucuronosyltransferase 2 family, polypeptide B17 (*UGT2B17*) detoxifies smoking-related metabolites. Differences among ethnic groups in *UGT2B17* are extremely high. Homozygous deletions of *UGT2B17* gene (*UGT2B17*-deletion) are a common copy number variant (CNV) among Japanese, but not a common CNV among Africans and Europeans. Thus, we examined Japanese patients with HNSCC to explore if *UGT2B17-*deletion and/or *p16 (+)* modify effects of smoking on *TP53-mutations* and affect relapse.

**Methods:**

We conducted a posthoc analysis of a prospective cohort. Polymerase chain reaction, immunohistochemistry, and direct sequencing were used to determine *UGT2B17*-deletion, *p16* (+), and detailed *TP53*-mutations, respectively.

**Results:**

*UGT2B17*-deletion was observed in 80% of this study population. For this 80%, *TP53-mutations* were significantly more common among smokers than non-smokers (*P* = 0.0016), but this difference between smokers and nonsmokers was not significant for the 20% with *UGT2B17*. In patients with *UGT2B17*-deletion and *p16 (+),* simultaneously, *TP53-mutations* were much more common among smokers than among non-smokers (81% versus 17%; *P* = 0.0050). Patients with both *UGT2B17*-deletion and disruptive *TP53-mutations* had higher relapse rates than other patients (hazard ratio, 2.22; 95% confidence interval, 1.30 to 3.80, *P* = 0.004) in a stepwise method.

**Conclusions:**

These results suggest that *UGT2B17*-deletion interacting with *p16* (+) may modify effects of smoking on *TP53-mutations* and may further interact with the disruptive *TP53-mutations* to raise relapse rates among Japanese patients with HNSCC.

## Background

Tobacco smoking is associated with 5 million deaths per year worldwide and is regarded as one of the leading causes of premature death [1]. Nicotine, a natural ingredient in tobacco leaves, is so addictive that people smoke habitually, which in turn results in exposure to a diverse array of carcinogens. Metabolites of nicotine, including cotinine and other compounds, are further catabolized and detoxified via CYP2A6 [2] and the UDP-glucuronosyltransferase (UGT) family of enzymes. One UGT gene, UDP-glucuronosyltransferase 2 family, polypeptide B17 (*UGT2B17)* enzyme decreases the abundance nicotine-related metabolites via glucuronidation [3]. Consequently, *UGT2B17* gene deletions may reduce detoxification rates of carcinogens in tobacco and tobacco smoke [4]. Therefore, this *UGT2B17*-deletion may increase an individual’s susceptibility to tobacco-related cancers, e.g., lung cancer [5].

Copy number variants (CNVs) of *UGT2B17* gene, known to vary greatly among ethnic populations; for example, homozygous deletion of *UGT2B17* (0 copy) is not a common CNV among Africans or Europeans e.g., 14% of Nigerians, but it is common among East Asian populations, e.g., 92% of Japanese [6]. Smoking is a major risk factor for head and neck squamous cell carcinoma (HNSCC) [7], by inducing oncogenic mutations of the *TP53* oncosuppressor gene [8] and of other genes [9,10]. In particular, disruptive mutations in *TP53* were associated with reduced survival in patients with HNSCC [11].

Therefore, we hypothesized that smoking may increase the risk of *TP53-mutations* among patients with homozygous for *UGT2B17* deletions (defined as “*UGT2B17*-deletion” in this study) to a greater extent than among patients with one or two copies of *UGT2B17* (defined as “*UGT2B17*-presence” in this study). Because *UGT2B17* deletion is common among Japanese, the power to detect interacting effects between smoking and *UGT2B17*-deletion on *TP53-mutations* can be enhanced by focusing on Japanese patients with HNSCC. In addition to *TP53-mutations*, overexpression of *p16*-protein [defined as “*p16* (+)” in this study] in tumors, which is encoded by *CDKN2A*, increases survival time in cases of oropharyngeal cancer [12,13]. We reported that heavy alcohol consumption triggered previously known and unknown somatic copy number alterations (SCNAs) including *CDKN2A*, but that smoking induced *TP53-*mutations [14]. Using this cohort of Japanese patients with HNSCC as post hoc analysis, we newly explored if *UGT2B17*-deletion modify effects of smoking on *TP53-*mutations, in combination with *p16* (+). Furthermore, we studied if combinations among *UGT2B17-deletion*, *p16* (+), and disruptive *TP53-*mutations affect cancer relapse.

## Methods

### Study design

We conducted a cohort study at Jikei University Hospital from March 2006 to November 2012. The study protocol was reviewed and approved by the Ethics Committee for Biomedical Research of the Jikei Institutional Review Board. The entire process of study design, data monitoring, and data analyses were performed in the Division of Molecular Epidemiology. Eligible participants were Japanese patients with HNSCC (oropharyngeal, hypopharyngeal, laryngeal, oral and nasal cancer) aged 20 years or older who had newly diagnosed or recurrent disease. A total of 262 patients provided written informed consent to participate in this study. Of these 262 patients, 28 patients were excluded because pathological diagnosis was not squamous cell carcinoma or because the primary tumor site was unknown. 27patient received in combination with chemotherapy or radiotherapy after surgery for close surgical margin and/or extracapsular spread of metastatic node. All of them were stage IV. Clinical data from the remaining 234 patients were used. Clinical information was obtained from clinical and surgical charts. Tumor node metastasis (TNM) classification and cancer stages were determined according to the 6^th^ Union for International Cancer Control TNM classification and stage groupings. Tumor grade with regard to cell differentiation was classified into three categories—well differentiated, moderately differentiated, or poorly differentiated—by a pathologist (M.I.). Of these 234 patients, nine patients were unknown of cell differentiation.

### Smoking and alcohol drinking

A history of current or past cigarette smoking was obtained based on a questionnaire completed by each patient at surgery. The age at which they started smoking and the number of cigarettes smoked per day was recorded. For past smokers, the age at which the patient ceased smoking was also recorded. The extent of previous smoking was quantified in pack-years (PYs); 10 PYs is any equivalent to smoking 1 pack including 20 cigarettes/day for 10 years (e.g., 2 packs/day for 5 years). Patients were classified as smokers if they had smoked at for least 10 PYs within the 20 years preceding diagnosis of HNSCC. Non-smokers were defined as patients who had never smoked, had not smoked in the 20 years preceding diagnosis, or smoked less than 10 PY prior to surgical resection of HNSCC. Of these 234 patients, two patients were unknown of smoking status.

The following three categories were used to classify patients based upon average daily alcohol consumption during the 20 years preceding diagnosis of HNSCC: 1) non-drinkers were defined as patients who did not consume alcohol or consumed less than one drink per day; 2) moderate drinkers were defined as patients who consumed at least one, but less than two, drinks per day, and 3) heavy drinkers were defined as patients who consumed two or more drinks per day. One drink was defined as containing approximately 10 g of alcohol, which is equal to 30 ml of hard liquor, 100 ml of wine containing 12% alcohol, or 360 ml of beer.

### Samples

With each patient’s consent, peripheral blood samples and tumor tissue were collected during the operation. QIAamp DNA Micro Kits 50 (Qiagen, Tokyo, Japan) were used to purify extracted DNA, and NanoVue plus (General Electric healthcare Japan, Tokyo, Japan) was used to measure DNA concentration in each sample; samples were then frozen at -80°C until use.

### Array-based comparative genome hybridization (CGH)

An Agilent Enzymatic Labeling Kit was used according to the manufacturer’s instructions to label 0.5 μg of genomic DNA for each CGH array. Labeled DNA was hybridized to an Agilent-022060 SurePrint G3 Human CGH Microarray 4x180K (Agilent Technologies, Inc., Santa Clara, CA, USA); the Agilent Microarray Scanner and Feature Extraction v.10.7.3.1 (Agilent Technologies), were used according to manufacturer’s instruction to scan probed arrays. Control DNA was obtained from one Japanese individual who is an author (MU) of this study. We focused only on previously reported SCNAs of *CDKN2A* [14] and on CNVs of *UGT2B17* that are associated with metabolism of nicotine [15]. The data described in this article have been deposited in NCBIs Gene Expression Omnibus (GEO) [16] and are accessible through GEO series accession number GSE47443.

### TaqMan Real-time PCR

We also performed real-time polymerase chain reaction (PCR) to confirm the microarray data. The TaqMan-based real-time PCR method for comparative quantification was performed with extracted DNA according to Life Technologies’ protocol. Genomic sequences of *UGT2B17* were used to generate the specific target sequence. Primers for *UGT2B17* (Taqman Copy Number Assays No. 186891217) and a probe for RNase P (Taqman copy number Reference Assay RNase P No. 4401631) were used (Life Technologies Corp.). Reactions (20 μL) were performed in 96-well plates using Brilliant III Ultra-Fast QPCR Master Mix, Reference Dye (30 nM), nuclease-free water (8 μL), DNA sample (1 μL), and *UGT2B17* primer (1 μL) (Applied Biosystems) or TaqMan Copy Number Reference Assay *RNase P* (1 μL); reaction mixtures were subjected to 40 cycles of 95°C for 3 min, 95°C for 10 s, and 60°C for 30 s. For the precise and accurate amplification of DNA, each assay with each primer pairs was run in duplicate. Comparative quantification was calculated using a sample from the same person (MU) who provided the control samples for the CGH array. A MX 3005P Real-Time QPCR System with Mx Pro Software version 4.10 (Agilent Technologies) was used to measure the product of each real-time PCR assay.

The method of measurement was based on the comparative cycle threshold (Ct) method for the target sequence (*UGT2B17*) and a reference sequence (*RNase P*). The *RNase P* gene was co-amplified with *UGT2B17* and served as an internal standard. The PCR amplification efficiencies of *RNase P* and *UGT2B17* were 100% and 99%; these were calculated by using the comparative ΔΔCt methods as described by Pfaffl et al. [17]. The fold changes in copy numbers of the gene were log2 transformed and determined to be gene positive or gene negative (over two copies or not). Finally, 97% of array results were consistent with real-time PCR.

### PCR to differentiate between one and two copies of UGT2B17

In 3% of samples, array and real-time PCR results were conflicted and could not differentiate between one and two copies of the *UGT2B17* gene, To determine the absence or presence of the *UGT2B17* gene, we further performed PCR as follows. Because a high level of sequence identity exists between the *UGT2B17* and *UGT2B15* genes, we used gene-specific PCR primers to distinguish *UGT2B17* from *UGT2B15* and to distinguish between one and two copies of the *UGT2B17* gene: Marker D (Forward primer 5’-TCACAAGTCAATCTCCCATCC-3’, Reverse primer 5’-CTGCAGAATATGTCAATAATTGGC-3’) is positive for one copy and two copies (100 bp), Marker J (Forward primer 5’-TGCACAGAGTTAAGAAATGGAGAGATGTG-3’, Reverse primer 5’-GATCATCCTATATCCTGACAGAATT-3’) is positive for only one copy (900 bp) [18,19]. PCR reactions were carried out in 25-μl mixtures containing 1 μg of genomic DNA, 2.5 μL of 10xLA PCR buffer II, 2 μL of dNTP (400 μM), 0.25 μL of LA Taq (Takara Bio Inc., Shiga, Japan), 18.25 μL of nuclease-free water, and 0.5 μL of each of the two primers (100 pmol/μL). Each reaction mixture was incubated at 94°C for 3 min and then subjected to 30 cycles of 94°C for 20 s, 60°C for 30 s, and 72°C for 90 s; each reaction was then incubated at 16°C until analysis.

### TP53-mutations

The quality or quantity of DNA samples from 14 patients was not adequate to assess *TP53* mutational status; therefore, only 234 samples were analyzed with regard to *TP53-mutations*.

Exons 2 thru 11 of the *TP53* gene were each independently amplified by PCR using purchased primers following the manufacturer’s protocol (NIPPON GENE Co. Ltd., Chiyoda-ku, Tokyo, Japan). Each resulting PCR product was cloned and then sequenced with the ABI PRISM 3700 Genetic Analyzer (Life Technologies Corp.). The following 10 single-nucleotide polymorphisms —V31I, P36P, P47S, P72R, R158R, R213R, V217M, P222P, T312S, and G360A—are reportedly each caused by a single nucleotide polymorphism [20], and thus excluded from total *TP53*-mutations. Disruptive *TP53-mutations* were defined as non-conservative mutations located inside the key DNA-binding domain (L2-L3 region) or as stop codons in any region [9]. Sites containing cytidine phosphate guanosine (CpG) dinucleotides were determined according to the database of WHO’s International Agency for Research on Cancer and based on the work by Petitjean et al. [21].

### *p16* immunohistochemistry

Formalin-fixed, paraffin-embedded tumor specimens were evaluated for *p16* overexpression with a rabbit monoclonal antibody that recognizes *p16* (Anti-*CDKN2A/p16INK4a* antibody [EPR1473]: Abcam Plc, Science Park, Cambridge, England). In this study, positive *p16*-protein expression (designated *p16* (+)) determined via immunohistochemistry (IHC) was defined as strong and diffuse nuclear, cytoplasmic staining or both in at least 70% of tumor cells. Any other pattern of *p16* expression was classified as *p16* (-).

### Statistical analysis

To evaluate significant differences between groups, the unpaired *t* test and the Mann-Whitney test were used to analyze ages and PYs, respectively. The chi-square test was used to assess categorical variables. Interaction effects between smoking and each of ten sub-groupings—age (< vs. ≥ 65 years), gender, drinking status, primary sites of tumor, tumor grades, stages, *UGT2B17-*CNV, *CDKN2A-*SCNA, *p16*-ICH, and *UGT2B17*-CNV and *p16*-ICH combined—were assessed with respect to any type of *TP53-mutations*; potential interactions were assessed by a *P*_*interaction*_ term. Then, for each sub-grouping, risks for any kind of *TP53-mutations* were compared between smokers and non-smokers using a risk ratio (RR) with a 95% confidence interval (95% CI).

In survival analyses, the time from surgery to relapse was used to calculate relapse-free rates. Patients were considered as “censored”, when follow-ups were stopped at the time of a patient’s death by causes other than HNSCC relapse or the last outpatient clinic visit. The Cox proportional hazard model was used to calculate each hazard ratio (HR) with a 95% CI. To distinguish significant prognostic factors from non-significant factors, a stepwise backward elimination method was applied to all 13 factors identified—age, gender, smoker (10PYs≤), heavy drinker, primary sites of tumor, *CDKN2A*-SCNA, *p16*-ICH, disruptive *TP53-mutations*, *UGT2B17*-deletion, interaction between disruptive *TP53-mutations* and *UGT2B17*-deletion, interaction between disruptive *TP53-mutations* and *p16* (+), stages, tumor grades— with a cutoff point of *P* = 0.05. The Kaplan–Meier survival curves were drawn based on relapse-free rates; log-rank tests were used to compare these rates differentiated by *p16* (+), *UGT2B17*-deletion and disruptive *TP53-mutations*. Each P < 0.05 was considered statistically significant. However, the Bonferroni correction was used to correct for multiple testing, and each pairwise interaction among the 10 subgroups was considered significant when *P*_interaction_ was less than 0.005. All statistical analyses were performed using STATA 13.1 (STATA Crop., College Station, TX).

## Results

### Patient characteristics

Patient characteristics were compared between non-smokers and smokers and between patients with wild-type *TP53* and those with any type of *TP53-mutations* in the primary tumors (Table 1). Tumors with *TP53-mutations* were significantly more common among smokers (67%) than among non-smokers (52%) (RR: 1.29, 95% CI: 1.00 to 1.65, *P* = 0.030), which we have already reported [14]. Men (*P* < 0.001) and alcohol-drinkers (*P* < 0.001) were also significantly more common among smokers than among non-smokers. Oral cancer was more frequent among non-smokers than smokers compared with other primary tumor sites (*P* = 0.030). Well differentiated histology was less common among smokers than non-smokers. Heterozygous and homozygous deletions of the *CDNK2A*-gene were significantly more prevalent among patients with *TP53-mutations* than those with wild-type *TP53* (*P* = 0.035). Additionally, we found that 80% of this study population harbored *UGT2B17*-deletions. However, non-smokers did not differ significantly from smokers with regard to *p16* (+) or *UGT2B17*-CNVs; similarly, patients with wild-type *TP53* did not differ significantly from those with *TP53-mutations* with regard to *p16* (+) or *UGT2B17*-CNVs.

**Table 1:** **Patient** ^*1^ **characteristics assessed based on smoking status and** ***TP53-mutations***

	Total	Smokers^*2^	Non-smokers^*2^	p-value	Mutant*TP53*	Wild-type*TP53*	p-value
		(N = 161: 69%)	(N = 71: 31%)		(N = 147: 63%)		
						(N = 87: 37%)	
Smoking status – PYs							
25%/50%/75%	0/25/40	25/40/46	0/0/0	<0.0001^*3^	8/30/40	0/20/40	0.085^*3^
Smokers – no. (%)	161 (69)	-	-	-	108 (74)	53 (61)	0.030^*4^
*TP53-mutations* – no. (%)	147 (63)	108 (67)^*7^	37 (52) ^*7^	0.030^*4^			
Age, years – yr. mean ± s.d.	63.2 ± 10.9	63.5 ± 10.2	62.6 ± 12.6	0.56^*5^	64.1 ± 10.4	61.6 ± 11.5	0.082^*5^
Men – no. (%)^*6^	187 (80)	152 (94)	33 (46)	<0.001^*4^	122 (83)	65 (75)	0.13^*4^
Drinking status – no. (%)^*6^				<0.001^*4^			0.043^*4^
Non-drinker	89 (38)	35 (22)	53 (75)		50 (34)	39 (45)	
Moderate drinker	74 (32)	64 (40)	9 (13)		55 (37)	19 (22)	
Heavy drinker	71 (30)	62 (39)	9 (13)		42 (29)	29 (33)	
Primary site of tumor – no. (%)^*6^				0.030^*4^			0.13^*4^
Oropharyngea	63 (27)	47 (29)	16 (23)		37 (25)	26 (30)	
Hypopharyngeal	64 (27)	47 (29)	16 (23)		49 (33)	15 (17)	
Laryngeal	29 (12)	24 (15)	5 (7)		17 (12)	12 (14)	
Oral	57 (24)	32 (20)	25 (35)		32 (22)	25 (29)	
Nasal	21 (9)	11 (7)	9 (13)		12 (8)	9 (10)	
Cell differentiation – no. (%)^*6^				0.023^*4^			0.94^*4^
Well differentiated	69 (31)	39 (25)	29 (43)		45 (31)	24 (29)	
Moderately differentiated	111 (49)	84 (54)	26 (39)		70 (49)	41 (50)	
Poorly differentiated	45 (20)	33 (21)	12 (18)		28 (20)	17 (21)	
Stages – no. (%)^*6^				0.12^*4^			0.97^*4^
I	12 (5)	11 (7)	1 (1)		8 (5)	4 (5)	
II	48 (21)	34 (21)	14 (20)		29 (20)	19 (22)	
III	48 (21)	28 (18)	20 (29)		30 (21)	18 (21)	
IV	124 (53)	87 (54)	35 (50)		79 (54)	45 (52)	
Anticancer therapy – no. (%)							
Radiotherapy ± Chemotherapy	27 (12)	22 (13)	4 (6)	0.13^*4^	15 (10)	12 (14)	0.41^*4^
*CDKN2A*-SCNAs – no. (%)^*6^				0.73^*4^			0.035^*4^
Norma	174 (77)	117 (76)	55 (80)		99 (72)	75 (86)	
Heterozygous deletion	39 (17)	29 (19)	10 (14)		29 (21)	10 (11)	
Homozygous deletion	12 (5)	8 (5)	4 (6)		10 (7)	2 (2)	
*p16* (+) – no. (%)	47 (20)	28 (17)	19 (27)	0.10^*4^	24 (16)	23 (26)	0.062^*4^
*UGT2B17* CNVs – no. (%)^*6^				0.60^*4^			0.95^*4^
Homozygous deletions: 0 copy	181 (80)	124 (81)	55 (80)		111 (80)	70 (80)	
Heterozygous deletion: 1 copy	42 (19)	28 (18)	14 (20)		26 (19)	16 (18)	
Normal: 2 copies	2 (1)	2 (1)	0 (0)	0.60^*4^	1 (1)	1 (1)	0.95^*4^

^*1^Smoking history was unavailable for two of the 234.

^*2^Non-smokers were defined as having a <10-PYs history; smokers were defined as having a ≥10-PYs history.

^*3^Mann-Whitney test was used to calculate the p-value.

^*4^*χ*^2^ test was used to calculate the p-value. ^*5^Student’s *t* test was used to calculate the --value.

^*6^Because of rounding, total values are not always 100%. ^*7^RR, 1.29; 95% CI, 1.00 to 1.65.

Then, we focused more closely on *TP53* status of tumors. Of the 234 tumor samples analyzed, 86 samples had no *TP53* mutation, 84 had one mutation, 27 had two mutations, 20 had three, 7 had four, 9 had five, and 1 had six. The frequencies of specific base-pair changes among these 234 patients were as follows: A:T > C:G, 1 (0.4%); A:T > G:C, 13 (5.6%), A:T > T:A, 5 (2%); G:C > A:T, 60 (26%); G:C > C:G, 19 (8%); G:C > T:A, 82 (35%). The frequencies of other types of mutations were as follows: deletion, 10 (4%); insertion, 4 (2%); nonsense, 63 (27%); missense, 69 (30%); frameshift, 14 (6%). In non-smokers, 9 in 37 (24%; 95% CI, 12 to 41%) *TP53-mutations* occurred at CpG sites, but in smokers, 13 in 108 (12%; 95% CI, 7 to 20%) did.

### Effects modifiers of smoking on *TP53-mutations*

Interactions between smoking and each of 11 variables—age, gender, alcohol drinking status, the primary sites of tumors, tumor grades, stages, the number of lymph node metastasis, *UGT2B17*-deletion*, CDKN2A*-SCNAs, *p16* (+), and a combination of *UGT2B17*-deletion and *p16* (+)—were assessed (Table 2). In variables of the primary sites of tumors, *CDKN2A*-SCNAs, *p16* (+), and a combination of *UGT2B17*-deletion and *p16* (+), interactions were analyzed except for HPV-positive patients. Smoking interacted significantly with four factors—stages, *UGT2B17*-deletion, *p16* (+), and the combination of *UGT2B17-*deletions and *p16* (+)—to induce *TP53-mutations*, but not with age (*P* = 0.55), gender (*P* = 0.22), drinking status (*P* = 0.90), primary tumor sites (*P* = 0.09), tumor grades (*P* = 0.30), the number of lymph node metastasis (*P* = 0.51) or *CDKN2A*-SCNAs (*P* = 0.08). Restricting to patients with *UGT2B17*-deletion, *TP53-mutations* were more prevalent among smokers than among non-smokers (*P* = 0.0016), but restricting to patients with *UGT2B17-*presence, differences between smokers and non-smokers were not significant. Similarly, restricting to patients with *p16* (+) tumors, smoking increased the risk of *TP53-mutations* up to 3.48-fold in comparison with non-smoking, but not in restricting to patients with *p16* (-) tumors. In restricting to patients with *UGT2B17*-deletion and had *p16* (+), smokers had significantly higher risk of *TP53-mutations* than did non-smokers (RR, 4.88; 95% CI, 0.80 to 29.6; *P* = 0.0050), but not in other combinations: *UGT2B17*-presence and *p16* (-), *UGT2B17*-deletion and *p16* (-), and *UGT2B17* presence and *p16* (+) (Table 2).

**Table 2:** **Effects modifiers of smoking on** ***TP53*** **-mutations in tumors** ^*1^

Outcome: any type of*TP53-mutations*	*P*_interaction_^*2^	Smokers N = 167	Non-smokers N = 65	RR	95% CI	p-value
Primary sites of tumor – no. (%)*6	0.09					
Oropharyngeal		26 (76)	4 (80)	0.96	0.59 to 1.54	0.86
Hypopharyngeal		37 (79)	11 (69)	1.15	0.80 to 1.64	0.42
Laryngeal		14 (58)	3 (60)	0.97	0.44 to 2.15	0.95
Oral		15 (48)	16 (67)	0.73	0.46 to 1.15	0.18
Nasal		10 (91)	1 (11)	8.18	1.28 to 52.4	0.0004
Stages – no. (%)	0.0019					
I		7 (64)	1 (100)	0.64	0.41 to 0.99	0.46
II		19 (56)	10 (71)	0.78	0.50 to 1.22	0.32
III		21 (75)	9 (45)	1.67	0.98 to 2.83	0.034
IV		60 (69)	17 (49)	1.42	0.98 to 2.05	0.035
*UGT2B17* CNVs^*5^ – no. (%)	0.0016					
*UGT2B17*-deletion: 0 copy		85 (69)	24 (44)	1.57	1.14 to 2.17	0.0016
*UGT2B17*-presence: 1 copy or 2 copies		16 (53)	11 (79)	0.68	0.44 to 1.05	0.11
*CDKN2A* SCNA^*3*6^ – no. (%)	0.08					
Normal: 2 copies		68 (65)	22 (51)	1.27	0.91 to 1.75	0.12
Heterozygous deletion: 1 copy		21 (78)	7 (70)	1.11	0.71 to 1.75	0.62
Homozygous deletions: 0 copy		6 (75)	4 (100)	0.75	0.50 to 1.12	0.27
*p16*-ICH in tumor^*4*6^ – no. (%)	0.043					
*p16* (-)		85 (68)	33 (66)	1.03	0.82 to 1.30	0.80
*p16* (+)		17 (77)	2 (22)	3.48	1.00 to 12.1	0.0043
*UGT2B17*-CNVs & *p16*-ICH – no. (%)^*6^	0.0080					
*UGT2B17*-presence & *p16* (-)		13 (57)	10 (91)	0.62	0.42 to 0.93	0.045
*UGT2B17*-deletions & *p16* (-)		67 (69)	21 (57)	1.22	0.89 to 1.66	0.18
*UGT2B17*-presence & *p16* (+)		2 (50)	1 (33)	1.50	0.23 to 9.80	0.66
*UGT2B17*-deletions & *p16* (+)		13 (81)	1 (17)	4.88	0.80 to 29.6	0.0050

^*1^Any type of *TP53*-mutations observed in the tumor genome was used as the outcome.

^*2^P_interaction_ was calculated as interaction effect between a factor and smoking on the risk of *TP53-mutations* in a tumor. With the Bonferroni correction, p < 0.005 was considered as statistically significant.

^*3^*CDKN2A* SCNA: Copy number alterations of *CDKN2A*, which encodes *p16*, were determined by CGH array.

^*4^*p16* overexpression in tumor samples was determined via ICH and classified as positive (+) or negative (-).

^*5^*UGT2B17* CNV: Copy number variants of *UGT2B17* were screened via CGH array and confirmed by real-time PCR and PCR to differentiate between one and two copies of *UGT2B17-*gene.

^*6^Interaction was analyzed except for HPV-positive patients.

### Prognostic factors

**Figure 1:**
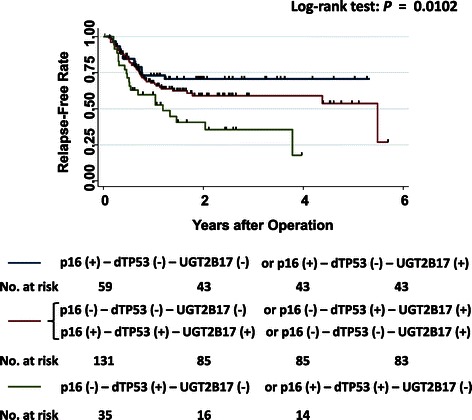
**Kaplan-Meier curves of relapse-free rates in 234 patients with HNSCC.** Differences in time until relapse were compared among combinations of *p16* (+) tumors, disruptive *TP53-mutations*, and homozygous *UGT2B17* deletions. The group of both *UGT2B17*-deletion and a disruptive *TP53-mutation* in the primary tumors is indicated as green-colored line, the group of both *p16* (+) and no disruptive *TP53-mutation* in the primary tumors is indicated as blue-colored line and the other groups are indicated as red-colored line. p16 (+): *p16*-positive tumor; p16 (-): *p16*- negative tumor; dTP53 (+): presence of disruptive *TP53*-mutations; dTP53 (-): no disruptive *TP53*-mutations or wild-type *TP53;* UGT2B17 (+): *UGT2B17*-deletion: homozygous deletion of *UGT2B17*; UGT2B17 (-): *UGT2B17*-presence: one or two copies of *UGT2B17*.

Using backward elimination for 13 candidate prognostic factors (Table 3), we found that disruptive *TP53-mutations* and *UGT2B17*-deletion interacted to significantly increase the risk of relapse (HR, 2.22; 95% CI, 1.30 to 3.80, *P* = 0.004); however, either *TP53-mutations* or *UGT2B17*-deletion alone did not significantly affect the risk. Notably, *p16* (+) was a better prognostic factor than *p16* (-) (HR, 0.53; 95% CI, 0.29 to 0.99, *P* = 0.047). Thus, we analyzed three grouping of the 234 patients based on combinations of three factors—*p16* (+) tumors, presence of disruptive *TP53-mutations*, and *UGT2B1*7-deletions. During a median follow-up period of 1.5 years (interquartile range, 1.0 to 2.5 years), relapse occurred in 89 of 232 patients (38%) in this study. Based on Kaplan-Meier curves, patients harboring both *UGT2B17*-deletion and a disruptive *TP53-mutation* in the primary tumors had the highest relapse rates among the three groups, and the group comprising patients with *p16* (+) tumors and lacking any disruptive *TP53-mutation* in the primary tumors had the lowest relapse rates (Figure 1). Relapse was occurred in 21 of 35 patients in the group of both *UGT2B17*-deletion and a disruptive *TP53-mutation* in the primary tumors (indicated as green-colored line in Figure 1), 16 of 59 patients in the group of both *p16* (+) and no disruptive *TP53-mutation* in the primary tumors (indicated as blue-colored line in Figure 1) and 48 of 131 patients in the other groups (indicated as red-colored line in Figure 1). We also analyzed overall survival by Kaplan-Meier curves. Although patients with *p16* (+) tumors and lacking any disruptive *TP53-mutation* in the primary tumors had the highest survival rates than the other groups (*P* = 0.0190, figure was not shown), there was no significant effect among these three factors; status of disruptive *TP53-mutation, p16* and *UGT2B17.*

**Table 3:** **Cox proportional hazard model as determined with backward eliminated via a stepwise method** ^*1^

Outcome: any type of*TP53-mutations*in a tumor	HR	95% CI	*p*-value
**Having both disruptive** ***TP53-mutations*** **and** ***UGT2B17*** **-deletions**	2.22	1.30 to 3.80	0.004
***p16*** **-positive tumor**	0.53	0.29 to 0.99	0.047
**Stage IV**	2.32	1.44 to 3.74	0.001
**Poorly differentiated tumor grade**	1.66	1.01 to 2.74	0.047

^*1^By backward elimination from age, gender, smoker (10 PYs ≤), heavy drinker, primary site of tumor, *CDKN2A-SCNAs*, *p16* (+), disruptive *TP53-mutations*, *UGT2B17*-deletion, interaction effect between disruptive *TP53-mutations* and *UGT2B17*-deletion, interaction between disruptive *TP53-mutations* and *p16* (+), stages, tumor grade of cell differentiation.

## Discussion

The prevalence of copy number variants (CNVs) of *UGT2B17* gene is quite different among ethnic populations. The frequency of *UGT2B17*-deletion was only about 10 to 15% among general Caucasian population or Caucasian with lung cancer [5,22]. In contrast, the frequency of *UGT2B17*-deletion among Japanese athletes was 74.5% in male and 60.2% in female [23] and 92% among those contributing to the Japanese HapMap [6]. We first confirmed that homozygous deletion of *UGT2B17* is highly prevalent among this cohort of Japanese patients. Of the 234 patients examined, 80% were homozygous for *UGT2B17*-deletions; 19% carried one copy of *UGT2B17*, and only 1% had two copies; these findings were within the range of previous reports for the Japanese or East Asian [6,23]. Therefore, Japanese patients with HNSCC constitute a valid study population for examination of the influence of *UGT2B17*-deletion on *TP53* mutation ratio and relapse rates.

We next found that 63% of all tumors had some type of mutation within exon-2 to exon-11 of *TP53*. There were significantly more *TP53*-mutant tumors among smokers than non-smokers, which we have already reported [14]. Moreover, the frequency of *TP53-mutations* at CpG sites was 2-fold higher among non-smokers than among smokers. These findings were consistent with previous findings from whole-exome sequencing studies [9].

Thirdly, we found a significant interaction effect between *UGT2B17*-deletion and smoking on *TP53* mutation rate (*P*_*interaction*_ = 0.0016). Specifically, restricting to patients with *UGT2B17-*deletion, *TP53-mutations* were significantly more common in tumors from smokers than those from non-smokers, but not for patients with *UGT2B17*-presence; to our knowledge, these and following findings have not been reported previously. *UGT2B17*-deletion may 1) reduce a person’s ability to detoxify smoking-associated metabolites, 2) allow cellular DNA to become exposed to high levels of carcinogens in cigarettes and cigarette smoke, and 3) raise the probability of DNA injury, and thus increase the risk of *TP53-mutations,* resulting in tumor development. Moreover, *p16* expression also interacted with smoking to increase the risk of *TP53-mutations*. Specifically, restricting to patients with *p16* (+) tumors, the frequency of *TP53-mutations* was 3.48-fold higher among smokers than non-smokers. Overexpression of *p16* can result from disruption of the negative feedback loop that normally operates among p16, cyclin-dependent kinases, cyclins, and phosphorylation of retinoblastoma protein; several causes—including human papillomavirus infection—can disrupt this loop [24], and such disruption may facilitate cell cycle progression and make cells more susceptible to the carcinogens in cigarettes and cigarette smoke.

We then assessed a combination of these two variables—*UGT2B17*-deletion and *p16* (+)—to assess potential interactions between them. Restricting to patients with *UGT2B17*-deletion and *p16* (+) tumors, *TP53-mutations* were significantly more common among tumors from smokers (81%) than those from non-smokers (17%) (RR, 4.88; 95% CI, 0.80 to 29.6; *P* = 0.0050). On the other hand, patients with *UGT2B17*-presence and *p16* (-) tumors, *TP53-mutations* were significantly less common among tumors from smokers (57%) than those from non-smokers (91%) (RR, 0.62; 95% CI, 0.42 to 0.93; *P* = 0.045). From these findings, together *UGT2B17*-deletion and *p16* (+) synergistically enhanced the risk of *TP53-mutations* occurring in tumors, because *UGT2B17*-deletion reduced the metabolism and detoxification of metabolites from cigarette smoke and *p16*-overexpression reflected abnormal cell cycle progression and increased cellular susceptibility to carcinogens.

In survival analyses, we confirmed that patients survival were significantly associated with stages, cell differentiation levels and the number of lymph node metastasis before adjustment; these results were consistent with previous studies. Then we analyzed using stepwise elimination and survival analysis by adjusting for stages, cell differentiation levels and others.

Fourth, we found a significant interaction between disruptive *TP53-mutations* and *UGT2B17*-deletion. Patients with *TP53*-mutant tumors and *UGT2B17*-deletion were more than twice as likely to relapse as all other patients; this finding was novel and striking. In contrast, patients with *p16* (+) tumors and wild-type *TP53* were half as likely to relapse as those with other patterns of tumor mutation; this finding was consistent with a previous finding about oropharyngeal cancer [12].

There were four main limitations in this study. Only two patients had two copies of *UGT2B17;* therefore, we mainly compared the effects of homozygosity with those of heterozygosity with regard to *UGT2B17*-deletions. Among 262 participants, *TP53-mutations* could not be measured in 28 samples, because of too small size of resected tumors to use for this study. Third is we analyzed relapse-free survival within the patients who had newly diagnosed or recurrent disease. It appears that one of the causes for no significant differences in the over survival may be the effect by the curative treatment to recurrence. Fourth is the most patients were advanced stage III to IV (74%) in this study. In spite of limited to the patients with early stage I to II, the patients harboring both *UGT2B17*-deletion and a disruptive *TP53-mutation* in the primary tumors had the highest relapse rates among the three groups using Kaplain-Meier curves (Log-rank test, P = 0.0071, figure was not shown).

## Conclusions

In conclusions, homozygous *UGT2B17*-deletion may interact with smoking and *p16*-protein expression to increase the risk of *TP53-mutations*, and may further interact with disruptive *TP53-mutations* to raise relapse rates among Japanese patients with HNSCC.

## Abbreviation

HNSCC: Head and neck squamous cell carcinoma
UGT2B17: UDP-glucuronosyltransferase 2 family, polypeptide B17
CNV: Common copy number variant
UGT: UDP-glucuronosyltransferase
SCNA: Somatic copy number alterations
TNM: Tumor node metastasis
PY: Pack-year
CGH: comparative genome hybridization
PCR: Polymerase chain reaction
CpG: Cytidine phosphate guanosine
IHC: Immunohistochemistry
RR: Risk ratio
95% CI: 95% confidence interval
HR: Hazard ratio
